# Antibacterial Activity of Propolis Extracts from the Central Region of Romania against *Neisseria gonorrhoeae*

**DOI:** 10.3390/antibiotics10060689

**Published:** 2021-06-08

**Authors:** Mihaela Laura Vică, Ioana Glevitzky, Mirel Glevitzky, Costel Vasile Siserman, Horea Vladi Matei, Cosmin Adrian Teodoru

**Affiliations:** 1 Department of Cellular and Molecular Biology, “Iuliu Hațieganu” University of Medicine and Pharmacy, 400012 Cluj-Napoca, Romania; mvica@umfcluj.ro; 2 Doctoral School, Faculty of Engineering, “Lucian Blaga” University of Sibiu, 550025 Sibiu, Romania; ioana_glevitzky@yahoo.com; 3 Faculty of Exact Science and Engineering, “1 Decembrie 1918” University of Alba Iulia, 510009 Alba Iulia, Romania; mirel_glevitzky@yahoo.com; 4 Department of Legal Medicine, ‘Iuliu Haţieganu’ University of Medicine and Pharmacy, 400012 Cluj-Napoca, Romania; cvsiserman@gmail.com; 5 Clinical Surgical Department, Faculty of Medicine, “Lucian Blaga” University, 550002 Sibiu, Romania; ateodoru77@yahoo.com

**Keywords:** *Neisseria gonorrhoeae*, ciprofloxacin resistance, antibacterial activity, propolis extracts

## Abstract

(1) Background: Sexually transmitted infections (STIs) are among the most common infections worldwide, many of these being caused by *Neisseria gonorrhoeae* (NG). Increased antimicrobial NG resistance has been reported in recent decades, highlighting the need for new sources of natural compounds with valuable antimicrobial activity. This study aims to determine the effect of propolis extracts on NG strains, including antibiotic-resistant strains. (2) Methods: First void urine samples from presumed positive STI subjects were harvested. DNA was extracted, purified, and amplified via PCR for the simultaneous detection of 6 STIs. The presence of the *dcmH*, *gyrA*, and *parC* genes was checked in the DNA samples from NG-positive patients. The antimicrobial activity of 5 aqueous propolis extracts from central Romania was investigated in vitro against some isolated NG strains. ANOVA tests were employed to assess differences and interactions between the inhibition zone for NG strains and propolis extracts. (3) Results: 7.07% of the patients presented NG infections, some strains being resistant or intermediate-resistant to ciprofloxacin. All propolis samples exhibited an antibacterial effect, including on resistant strains. (4) Conclusions: Statistical analysis demonstrated that the diameter of the inhibition zone was influenced both by the NG strain type and the source of the propolis extracts.

## 1. Introduction

*Neisseria gonorrhoeae* (NG), the gonococcus, is a Gram-negative bacterium that causes gonorrhea, one of the most common sexually transmitted infections (STI), with an estimated number of 87 million new cases in 2016 [1]. Social determinants of health, such as socioeconomic status, the presence of individuals engaged in unprotected sex at early ages, and the lack of prevention education may contribute to the burden of gonorrhea in a community [2].

For the past 70 to 80 years, gonorrhea has been treated successfully with the help of antimicrobials. Nonetheless, at present, gonococcal infections are most common because NG was able to develop resistance to several classes of antibiotics [3,4]. In recent years, NG has become less susceptible to several antibiotics such as sulfonamides, penicillins, tetracyclines, fluoroquinolones, and even cephalosporins [5]. The Gonococcal Antimicrobial Resistance Surveillance Programme has shown high rates of quinolone resistance, increasing azithromycin resistance, and emerging resistance to extended-spectrum cephalosporins. The development of drug resistance, particularly in the case of gonorrhea, has a major negative impact on reducing STIs worldwide [6].

Fluoroquinolone-resistant isolates were traditionally prevalent in Asia, but recently have been isolated with increased frequency in the United States. In response to this threat, many countries have revised their recommendations for the treatment of gonorrhea [7]. First-line therapy was changed to single-dose oral administration of cefixime because of the high prevalence of fluoroquinolone-resistant NG strains observed in the last decade. Intensely used previously, ciprofloxacin is still recommended in the treatment of presumptive gonorrhea patients allergic to cephalosporins [8].

The use of molecular assays for gonococcal antimicrobial resistance surveillance is particularly suited for fluoroquinolones, in which mutations occur at defined bases in the quinolone resistance-determining regions (QRDRs) of the NG *gyrA* and *parC* genes. Well-characterized QRDR mutations correlate with decreased gonococcal antimicrobial susceptibility to fluoroquinolones (MIC ≥1 µg/mL) [9].

Multiple research studies focused on alternative approaches to treat STIs. Herbal treatments were found to have several benefits as an alternative drug. For instance, *Eucalyptus,* which contains essential oil, exhibits antimicrobial properties against certain bacteria. The antimicrobial effects of this plant are generally greater on Gram-positive bacteria. In some cases, eucalyptus has been prescribed for the treatment of gonorrhea [10,11]. In addition, Soma (leaves, tree bark, roots, and fruit) is traditionally known as one of the effective herbal drugs that help fight certain diseases, including gonorrhea [10,12]. Likewise, the cannabis root is used to help treat this infection [13].

Several remedies for gonorrhea are mentioned by other studies, including garlic [14], apple cider vinegar [15], goldenseal [16], *Echinacea* spp. [17], *Aloe vera* [18], olive leaf extract [19], guyabano fruit [20], and parsley and celery [21].

There are studies that have shown that due to their antibacterial effect, bee products can be used in the treatment of a wide range of human diseases, including infections [22,23]. The *Snodgrassella avli* bacterium identified in the gut microbiota of honeybees has the potential to provide protective immunity against gonorrhea [24]. Although its actual effect on NG bacterium is not clear, raw honey dissolved in warm liquids is used in traditional medicine to relieve sore pain in gonorrhea-infected throats [25,26].

Propolis or “bee glue” is a complex mixture produced by the *Apis mellifera L*. bees, consisting of a resinous substance harvested from plant exudates and buds and mixed with wax and bee enzymes [27,28]. The chemical composition of propolis varies due to its geographical origin, plant source, hive, season, etc. [29,30]. Its composition includes resin, waxes, essential oils, pollen, and various other organic compounds, all in all more than 50 constituents [31,32,33,34]. Propolis has attracted the interest of researchers for its pharmaceutical properties, including its antioxidant, anti-inflammatory, and antimicrobial potential [35,36]. Pinocembrin, chrysin, pinobanksin, apigenin, and kaempferol were the predominant flavonoid derivatives identified in Polish ethanol extracts of propolis samples exhibiting a strong antioxidative action in vitro against bacteria and fungi, while the p-coumaric, ferulic, and caffeic acids were the main polyphenolic components identified there [37].

This study aimed to evaluate the antimicrobial potential of propolis extracts from several central Romania counties. They were tested against four NG strains: three isolated from STI patients and a reference one. This research also analyzed the correlations between the extracts’ origin and their antimicrobial activity, determined by measuring the diameter of the inhibition zones on the NG strains.

## 2. Results

### 2.1. Characterization of the Raw Propolis Extracts

The propolis samples collected from various Romanian counties were analyzed in terms of aspect, water content and activity, total ash percentage, and phenolic and flavonoid content (Table 1). Flavones and aromatic acids were identified in all five samples.

Table 1 data are expressed as means ± standard deviation of triplicate assays.

### 2.2. NG Detection in Urine Samples

A total of 200 of the 622 patients aged 17 to 75 years included in the study were found to be positive for STIs, 44 of these (7.07% of the total) presenting the corresponding NG DNA (the third most frequently detected bacterium after Chlamydia trachomatis and Ureaplasma urealyticum). NG was identified in 16 cases of simple infections and in 28 other samples in association with one or more STI agents.

### 2.3. dcmH, gyrA, and parC Gene Detection

The dcmH gene amplicons were spotted in all the samples collected from gonorrhea-infected patients, confirming the accuracy of both PCR methods employed in the study.

The gyrA and parC genes were amplified in 34 samples. The two genes were also highlighted by the positive control sensitive to ciprofloxacin (the ATCC 49,226 strain), confirming that the primers were correctly designed. The gyrA gene was not amplified in 6 samples presenting only the parC and dcmH genes, while 4 samples generated neither gyrA nor parC amplicons.

### 2.4. Assessment of Antimicrobial Activity of Propolis Samples

The antimicrobial properties of analyzed aqueous extracts of propolis samples are shown in Table 2. All aqueous extracts exhibited antimicrobial activity against the strains used.

The diameter of the inhibition zones for the strains tested with propolis extracts varied from 27 to 42 mm, in some cases exceeding the inhibition zones induced by ciprofloxacin. Sample S1 exhibited the greatest antibacterial effect (mean diameter 39.75 mm), the lowest being observed in sample S5 (30.75 mm).

The results for the NGI strain were close to those observed on the reference strain. All inhibition zone diameters exceeded 27 mm, including the ciprofloxacin-resistant NGIII strain. The latter, exhibiting an inhibition zone diameter of 20 mm against ciprofloxacin, was also the least inhibited by the propolis samples.

#### Minimum Inhibitory Concentration (MIC) of Different Types of Propolis

The propolis inhibitory effect in 1/1 and 1/4 dilutions was observed in all samples except for NGIII (in S5 for the 1/4 dilution). Lower dilutions (1/16, 1/32) produced partial effects in certain bacterial strains only. The results of the MIC values are presented in Table 3.

### 2.5. Statistical Analysis

The bifactorial dispersion analysis tested the simultaneous influence of two independent variables: the diameter of the inhibition zones for different microbial NG strains and propolis extracts from different Transylvanian counties. The results of the bifactorial dispersion analysis for the values presented above are illustrated in Table 4.

The column average values were influenced by the propolis origin, while the row averages were influenced by the selected strain. Because F_col_ > F_0.05_ = 3.26, the hypothesis that the columns’ mean values were equal was rejected and it was concluded that the origin of the propolis samples used influenced the diameter of the inhibition zones. It was also concluded that the gonococcal strains influenced the inhibition diameter at the chosen significance threshold α = 0.05, because F_row_ > F_0.05_ = 3.49. The factorial analysis of variance (ANOVA) illustrated that both the gonococcal strains and the origin of propolis extracts influenced the diameter of the inhibition zones.

## 3. Discussion

The large number of patients positive for NG (22% of the positive patients) indicates that infection with this bacterium remains one of the major causes of STIs in our country. An accurate diagnosis of gonorrhea is needed in order to prevent severe complications and to control transmission, especially in asymptomatic infections.

Recent years have seen extensive usage of nucleic acid amplification tests (NAATs), in particular for detecting carriers. These genetic tests enable establishing a diagnosis for gonococcal urethritis or cervicitis with a sensitivity of up to 99%. NAAT testing was also proven to be efficient in typing NG strains including non-cultured specimens [38]. For NG detection, we used a multiplex PCR (Seeplex^®^ STD6 ACE Detection) method with high specificity and sensitivity able to detect the presence of several STI pathogens in the same sample, useful since these often associate and may present similar symptoms. As a result, in our 200 positive samples, we found 46 associations of 2 to 5 STI pathogens, 28 of them involving NG.

In recent years, due to the excessive use of antibiotics, microorganisms have developed increased resistance to them [39]. Gonorrhea has quickly developed resistance to all but one class of antibiotics and half of all infections are resistant to at least one antibiotic. Because gonorrhea spreads easily, some men and most women do not present symptoms and may be unaware that they are infected. Untreated gonorrhea can cause serious and permanent health problems in women and men, including ectopic pregnancy and infertility, or cardiovascular and neurological problems when spreading into the blood vessels [40].

The continually increasing resistance to ciprofloxacin, a drug not used in the routine treatment of gonococcal infections, highlights the ongoing selective pressure generated by the use of antibiotics. There is an urgent need for new approaches, including collaborative efforts to determine how culture-based tests and NAAT may be combined or complemented to strengthen antimicrobial resistance surveillance [41].

A previous study [8] demonstrated that simultaneous non-generation of *gyrA* and *parC* amplicons consistently predicted the presence of ciprofloxacin-resistant gonococci and characteristic point mutations in the *gyrA/parC* QRDRs were found in DNA amplified from those extracts that failed to produce *gyrA/parC* amplicons. In view of these hypotheses, we tested the presence of *gyrA* and *parC* amplicons in order to select those NG strains able to provide the greatest possible variance in terms of antibiotic resistance (these determinations being part of another study of ours). The presence of the NG-specific *dcmH* gene served to confirm NG infections.

According to the Centers for Disease Control and Prevention (CDC) and to the Clinical and Laboratory Standards Institute (CLSI) on interpretive criteria for NG susceptibility testing [41], NG strains can be considered sensitive to ciprofloxacin if the diameter of the inhibition zone is at least 41 mm and resistant if it is less than 27 mm, with the strains being considered intermediate resistant between the two limits. Of the strains we isolated, one (NGI) was sensitive to ciprofloxacin, the diameter of the inhibition zone being 41 mm; another one (NGII) was intermediate–resistant, while the NGIII strain, with a diameter of 20 mm, can be considered resistant to ciprofloxacin. For other antimicrobial agents, the diameters of inhibition zones for which they are considered resistant or intermediate resistant are much smaller (e.g., for spectinomycin, less than 18 mm) [42]. In our case, the diameter of the inhibition zones for all strains tested with propolis extracts was more than 27 mm (see Table 2).

The results of this study indicate that propolis extracts from all five Transylvanian counties of origin present antimicrobial activity against all NG strains, sometimes even stronger than that of the antibiotic tested. The propolis extracts were also active at lower concentrations (see Table 3), MIC values of the S1, S2, and S4 samples being 6.25 µg/mL for all strains. Another study on propolis highlighted a higher activity against most Gram-negative bacteria with MIC in the range of 6.25 µg/mL to 500 µg/mL [43].

Since antiquity, physicians have expressed concerns about the effect of gonorrhea on infertility in both sexes. Treatments for gonorrhea in the medieval world included honey in water or milk [44]. Another bee product, propolis, was tested in our study. The presence of quercetin, with strong antimicrobial activity, and the high concentration of polyphenols and flavonoids, explain the propolis activity. It depends on its chemical composition, which in turn depends on the area of origin of the bee product across the different Transylvanian counties of origin, the bee species processing it, and the strain it is tested on. It was argued that the chemical composition of propolis varies considerably according to the geographic area of origin [45]. The propolis contains various secondary plant metabolites, which differ in concentration depending on season, geographic origins of the collection, and the proximity of a beehive to particular plant sources [46]. Another study found variations in the chemical composition, and consequently, in the biological activity of the propolis, that were associated with its subtype (brown, green, red, yellow, etc.) and geographical place of origin [47].

Another study on aqueous propolis extracts originating from the same region demonstrated that all propolis extracts presented higher antimicrobial activity on the studied strains, Gram-positive, Gram-negative, or fungi (*Escherichia coli, Staphylococcus aureus, Bacillus cereus, Pseudomonas aeruginosa*, and *Candida albicans*) than that of the tested antibiotic, ciprofloxacin [48].

Regarding the characterization of raw propolis samples (see Table 1), the water content below 8% in all our samples is in line with the required humidity standards for the brown variety [49]. Correspondingly, the samples with greater humidity presented a higher water activity [50]. The water activity and humidity can be used to predict microbial growth, microbial stability, conservation, and the occurrence of chemical reactions in the products [51].The total ash percentage in propolis can spot any adulteration [32] and the values obtained in this study are similar to those of Devequi-Nunes et al. (2018) [50] and Machado et al. (2016) [52].

More than 21 components were identified in propolis samples, including flavonoids (flavones, flavonols, and flavonones), aromatic (benzoic, cinnamic, feluric, and caffeic) acids, valine, cinnamyl and benzyl alcohols, esters of the benzoic, cinnamic and coumaric acids, as well as coumarinic and terpenoid derivatives. Aromatic esters are a fraction of the support of microbial action [53,54,55], their presence or absence explaining inconsistencies in regard to the antimicrobial activity of different propolis types [56,57,58].

Significant differences were found between the physico-chemical parameters of the propolis samples collected from various counties, especially in terms of phenolic and flavonoid content. Such variation can be explained by the type of flora, the region, and the period of sampling [32,59].

In propolis, the main chemical classes with antimicrobial properties are flavonoids and phenolic compounds [60]. In our study, the content of the phenolic compounds varied from 134.7 ± 4.09 (S3) to 203.3 ± 7.28 mg GAE/g (S1), while the flavonoid content ranged from 69.23 ± 0.04 (S5) to 90.54 ± 0.06 mg QE/g (S1). Flavones and aromatic acids were identified in all our samples. Arguably, a correspondence between the antimicrobial activity of the analyzed propolis samples (expressed by the diameter of the zones of inhibition) and the flavonoid/phenolic content was observed.

Studies on compounds isolated from the aqueous and hydro-alcoholic extracts of propolis (e.g., flavononol, sakuranetin or gallic acid) from Brazilian native bees highlighted that these components also presented antimicrobial activity against STI-inducing bacteria without a cell wall (*U. urealyticum*, *Mycoplasma hominis*, and *Mycoplasma genitalium*) and may constitute an undervalued alternative source of compounds with biological activity [61]. Red propolis from Brazil and Cuba exhibited antimicrobial activity against another *Neisseria* species (*N. meningitidis*) [43], as did a Bulgarian propolis [62].

A study on the antibacterial activity of various extracts of propolis harvested during winter and spring from several locations of Portugal, against Gram-negative and Gram-positive bacteria, concluded that all tested bacterial strains presented susceptibility to diluted propolis extracts in a dose-dependent manner. Two propolis samples collected at springtime exhibited higher antibacterial activity compared to samples harvested at wintertime [63]. Our statistical analysis identified several connections between the origin of propolis extracts, the microbial strains used, and antimicrobial activity. Variance analysis demonstrated that both NG strains and the origin of the propolis extracts used bear a certain influence on the diameter of inhibition zone.

We focused on propolis as a natural antibiotic and as a possible alternative drug. This is the first approach on the biological activity of Romanian propolis from the Transylvania region against NG and evidences its potential uses to combat bacterial infections. Further studies on varied propolis sources harvested at different times during the year are needed to observe variations of the antimicrobial activity of samples with the same origin and to standardize propolis extracts in terms of activity and composition to guarantee their quality and safety of use.

## 4. Materials and Methods

### 4.1. Propolis Sampling

**Figure 1:**
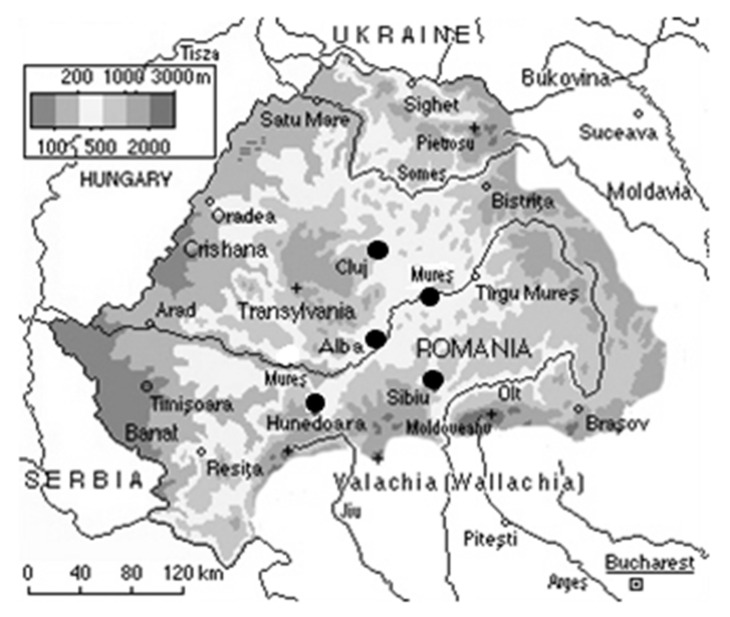
Romanian map highlighting the propolis sampling areas (Alba—S1, Cluj—S2, Hunedoara—S3, Sibiu—S4, Mureș—S5).

The antimicrobial properties of the propolis samples obtained on harvesting day from five Transylvanian counties during 2014 (see Figure 1) were tested against some NG strains.

A quarter liter of distilled water was added to 50 g of finely chopped propolis weighed on a Kern ABT120-5DNM analytical balance (Kern & Sohn GmbH, Balingen, Germany) and the mixture was refluxed for one hour in a round bottom flask provided with a condenser. The heterogeneous system was centrifuged at 4500× *g* on a Centra CL2 device (Thermo Fisher Scientific Inc., Waltham, MA, USA), coarsely filtered through a vacuum filter (Merck KGaA, Darmttadt, Germany), re-centrifuged at 4000× *g* and re-filtered in vacuum through a low porosity surface, then maintained at water boiling point until 80% of the initial mixture was evaporated [64]. The aqueous extracts of propolis thus obtained were stored in a chilly, dry, dark storage space until their analysis. In testing their antimicrobial activity, we used samples of 0.1 g/mL concentrations.

### 4.2. Physico-Chemical Analyses of the Propolis Samples

Several physico-chemical parameters were analyzed: the water content and activity, the mineral content, the presence of flavones and aromatic acids, and the assessment of the phenolic and flavonoid content in the propolis samples. Each determination was triplicated, and the average value was reported.

#### 4.2.1. Assessment of Water Content (H)

Propolis extract samples of 3.0 g were repeatedly dried in an oven at 135 ± 2 °C for one hour, then cooled in a desiccator and weighed until a constant weight was reached [65].

#### 4.2.2. The Water Activity (a_w_)

The water activity (a_w_) of propolis samples was recorded at 25 °C using the Aquaspector AQS-2-TC (Nagy Messsysteme GmbH, Gäufelden, Germany).

#### 4.2.3. Assessment of Total Ash

Propolis was dried at 550 °C for 1 h, cooled in a desiccator, and weighed. A measure of 3.0 g of sample was then introduced into a porcelain crucible and calcined at 550 °C for 12 h until white ashes were obtained [66,67].

#### 4.2.4. Qualitative Identification of Flavones’ Presence

Five grams of propolis powder were homogenized with 20 mL of ethanol 96% for 3 h. The extract was filtered and heated on a sand bath until a viscous mass was obtained, then 5 g of borax and 10 mL of distilled water were added and stirred for 10 min, the resulting cloudy liquid being filtered afterwards. Two points 15 cm apart were marked on a horizontally placed strip of filter paper and a few drops were pipetted onto them to produce two yellow spots. The presence of flavones was confirmed if placing 2–3 crystals of uranyl nitrate and distilled water on one of the spots turned the color into reddish brown, while adding 2–3 crystals of ferric sulfate and distilled water on the other spot changed its yellow color into gray [68].

#### 4.2.5. Identification of Aromatic Acids

Diluted sulfuric acid was added to 5 mL of the final solution prepared to identify the flavones (4.2.4.) until a precipitate resulted, then 10 mL of diethyl ether was added to the heavily stirred suspension. The upper layer containing aromatic derivatives in the extraction ether was collected in a clean glass beaker. The lower aqueous layer was subjected to the same operation and the new upper extraction layer was again collected in the same beaker. The content of the beaker was filtered on anhydrous Na_2_SO_4_ and the dehydrated solution was evaporated to dryness, then 10–20 drops of 2n NaOH and a few drops of 1n KMnO_4_ were added to the residue. Light heating allowed the identification of the benzoic aldehyde (specific smell of bitter almonds) and cinnamic aldehyde (giving off a distinct cinnamon smell) [68].

#### 4.2.6. Quantification of the Phenolic Compounds (the Folin–Ciocalteu Method)

An extract containing 0.5 g of raw propolis and 15 mL of ethanol was homogenized at 500 rpm for 30 min, then filtered and stored in the dark. To 500 μL of ethanolic propolis extract, a similar amount of Folin–Ciocalteu reagent was added. After a pause of 2 min, 2 mL of 10% sodium carbonate solution and distilled water were added for a final volume of 50 mL. Spectrophotometric measurements (Lambda 20—Perkin Elmer UV/VIS, Washington, DC, USA) were carried out at 765 nm with distilled water serving as blank. The results reflecting the concentrations of total phenolics were compared to a standard curve of gallic acid (mg GAE/g) under the same conditions. All analyses were triplicated [69,70]

#### 4.2.7. Determination of Flavonoid Content (Aluminum Chloride Colorimetric Method)

An extract containing 1 g of raw propolis and 25 mL of 95% ethanol were homogenized at 200 rpm for 24 h and then filtered. The filtrate was adjusted to 25 mL with 80% ethanol and kept in the dark [71]. Then, 10 mg of quercetin was dissolved in 80% ethanol and then diluted to elaborate a standard curve. The diluted standard solutions (0.5 mL) were separately mixed with 1.5 mL of 95% ethanol, 0.1 mL of 10% aluminum chloride, 0.1 mL of 1M potassium acetate, and 2.8 mL of distilled water. The flavonoid content was assessed using a spectrophotometer (Lambda 20—Perkin Elmer UV/VIS) at 415 nm. The quantity of flavonoid content was expressed as quercetin equivalents (mg QE/g). All the analyses were triplicated [69,72].

### 4.3. The Selection of NG Strains in View of Their Antimicrobial Activity

#### 4.3.1. Patients and Specimens

A total of 622 subjects presenting STI symptoms such as dysuria, hematuria, pollakiuria, vaginal/urethral secretions, or lower abdominal pain, combined or not with fever, as well as at-risk asymptomatic individuals involved in unprotected sex with multiple partners were tested for STIs. The study was conducted between January 2014 and September 2019 and implied harvesting first void urine samples of 30 to 50 mL to be collected in sterile containers. Precautions were taken to make sure that the patients had not urinated in the 4 h preceding harvesting and had not been subjected to antibiotic treatment for at least a couple of weeks before being tested.

#### 4.3.2. DNA Extraction

The urine samples were aliquoted and centrifuged for 15 min at 15,000× *g*, the resulting DNA pellet being suspended in phosphate-buffered saline (PBS). For its extraction, we used a MasterPure™ Complete DNA and RNA Purification Kit (Epicentre Biotechnologies, San Diego, CA, USA), according to producer recommendations. The DNA was further suspended in nucleotide-free water. A Pearl Nanophotometer (Implen GmbH, Munich, Germany) was employed in determining the DNA concentration and purity. Automatic readings against reference nucleotide-free water identified extracts of inappropriate purity for which the DNA purification sequence was repeated.

#### 4.3.3. DNA Amplification for NG-positive Samples Detection

Traditional PCR was employed for DNA amplification in the process of identifying NG-positive samples, making use of a Biometra thermal cycler (Analytik Jena, Jena, Germany) and a Seeplex^®^ STD6 ACE Detection kit (Seegene, Seul, Korea). The PCR mix (17 μL) contained primer pairs for six STI agents including NG, along with internal control template and primers for internal control, which were added to 3 μL of DNA extracts. Following the 40 cycle PCR amplification protocol (30″ template denaturation at 94 °C, 90″ primer annealing at 63 °C, 90″primer extension at 72 °C), DNA fragments were separated by 2% agarose gel electrophoresis containing ethidium bromide, in the running buffer.

#### 4.3.4. DNA Amplification to Detect NG *dcmH*, *gyrA,* and *parC* Genes

For the detection of the *gyrA, parC*, and the specific *dcmH* genes amplicons in the NG positive samples, in order to select suitable antibiotic-sensitive/resistant or intermediate-resistant NG strains, we used a modified version of previously published sequences [8,9]. The sequences of the primers are presented in Table 5.

We used 6 μL of specific primers (containing primer pairs for *gyrA*, *parC*, and *dcmH* gene) for 2 μL of NG DNA extract from NG-positive samples, the reaction mix also including 25 μL MyTaqTM Red Mix (Bioline, Toronto, Canada) and 17 μL water (ddH_2_O). DNA extracted from a NG ATCC 49,226 strain (Thermo Fisher Scientific Inc.) served as positive control.

The amplification protocol was optimized in successive attempts, the final recipe including 35 cycles (15″ denaturation at 95 °C, 15″ annealing at 58 °C, 10″ extension at 72 °C) following a one-minute template denaturation at 95 °C. The amplicons were then separated via 2% agarose gel electrophoresis and visualized in UV due to ethidium bromide staining.

#### 4.3.5. NG Strains Isolation

We isolated 3 NG strains promising to provide maximal variation in order to test the antibacterial properties of the propolis samples: one identified in a patient from whose sample both the *gyrA* and *parC* genes were amplified (NGI), a second one from a patient in whom only *parC* amplicons were spotted (NGII), and a third one from a patient lacking both *gyrA* and *parC* genes amplicons (NGIII).

Aliquots of first void urine samples from the three patients were centrifuged for 15 min at 15,000× *g* then the pellet was re-suspended in 1 mL sterile normal saline and the sediment was inoculated into chocolate agar medium plates (Thermo Fisher Scientific Inc.). The plates were incubated at 35 °C to 36.5 °C in a 5% carbon dioxide-enriched atmosphere for 24 h. Several NG-resembling colonies were re-inoculated on medium chocolate agar and the purity of the cultures obtained was tested microscopically (for the presence of Gram-negative diplococci only) and biochemically (the oxidase test, *Neisseria* spp. being one of the oxidase-positive species).

A loop of gonococcal reference strain ATCC 49,226 was re-hydrated in about 0.5 mL of sterile normal saline. The suspension of re-hydrated organisms was inoculated onto plates of chocolate agar medium and incubated 24 h at 35 °C to 36.5 °C in an enriched carbon dioxide-atmosphere.

### 4.4. Antimicrobial Activity

To assess the antibiotic susceptibility of the selected NG strains (see Section 4.2.5), we used the disk diffusion method according to CLSI-recommended procedures.

Colonies isolated from cultures on chocolate agar medium were suspended in 1.0 to 2.0 mL of Mueller–Hinton broth (Merck KGaA). The turbidity of the cell suspension was measured using a McFarland Densitometer (Mettler Toledo, Columbus, OH, USA) and adjusted by adding additional Mueller–Hinton broth or microorganisms, as required, until the turbidity of the suspension was equivalent to the turbidity of a 0.5 McFarland BaSO_4_ standard. GC agar base medium plates (Thermo Fisher Scientific Inc, Waltham, MA, USA) containing 1% (*v/v*) IsoVitaleX (Thermo Fisher Scientific Inc.) were used in the inoculation process. The entire surface of each plate was inoculated using a sterile cotton swab moistened in the prepared culture suspended in the Mueller–Hinton broth to ensure a uniform, confluent growth. The inoculated plates were rested for 3 to 5 min at room temperature to allow the moisture from the inoculum to absorb into the medium.

A propolis extract (50 µL) of 0.1 g/mL in concentration (sampled according to Section 4.1) was added to ~6 mm filter paper disks prepared in the laboratory. Disks impregnated with 5 µg ciprofloxacin (Bio-Rad, Hercules, CA, USA) served as controls.

When the surface of the medium was dry, the impregnated disks were applied to the surface of the medium and tamped gently with a sterile loop to ensure that they were in full contact with the agar surface. All disks were applied at approximately the same distance from the edge of the plate and from each other. The plates were then incubated at 35 °C to 36.5 °C in a 5% enriched CO_2_ atmosphere for 20 to 24 h (lid side down).

The diameter of each inhibition zone was measured with a DIN 862 ABS digital caliper (Fuzhou Conic Industrial Co. Ltd., Fujian, China) with +/−0.01 mm accuracy. The diameter of the paper disk was not subtracted from the result.

#### Minimum Inhibitory Concentration (MIC) of the Propolis Samples

The MIC values were determined using the dilution method. Graded doses (*v/v*) of the five propolis samples were dissolved in sterile deionized water to 1/1, 1/4, 1/8, 1/16, 1/32, and 1/64 final volume dilutions. The antimicrobial activity was assessed by the disk diffusion method as described earlier (see 4.4). GC agar base medium plates containing 1% (*v/v*) IsoVitaleX were used in the inoculation process. The filter paper disks were impregnated in propolis extract aliquots (50 µL of the above mentioned dilutions) and the disks were applied to the surface of the medium, the diameters of the inhibition zones being measured with a DIN 862 ABS digital caliper.

### 4.5. Statistical Analysis

Two-way analysis of variance (ANOVA) tests were used to assess differences and interactions between the diameter of inhibition zones for different NG strains and propolis originating from different Transylvanian counties [73]. The significance level for the entire study was set at α = 0.05. The two-way ANOVA was performed using Origin 8.0 (OriginLab Corp., Northampton, MA, USA) software.

## 5. Conclusions

The study demonstrated that NG remains an important cause of STIs in Romania as the existence of antibiotic-resistant strains was documented here.

All aqueous propolis extracts exhibited antimicrobial activity against the reference and the studied NGI–III strains. The propolis samples from Alba and Cluj counties exhibited higher antimicrobial activity. The reference strain, along with the NGI and NGII strains, were more sensitive to the bactericidal activity of the propolis samples. The NGIII strain presented a lower resistance to the propolis extract, while ciprofloxacin presented a higher antimicrobial activity against the reference and NGI strains and lower activity against the NGIII strain. Statistical analysis identified correlations between the origin of propolis, selected gonococcal strains, and antimicrobial activity. A correlation between the content of flavonoids and phenolic compounds and the antimicrobial activity of the analyzed propolis samples was observed.

This study was the first to evaluate the antimicrobial effect of propolis extracts produced in Transylvania against NG, arguing its potential use in naturistic medicine.

## Figures and Tables

**Table 1:** Physico-chemical parameters of the propolis samples.

Sample/ Shade of Brown	H (%)	a_w_ (%)	Total Ash Content (%)	Phenolic Compounds (mg GAE/g)	Flavonoids (mg QE/g)
S1—pale	7.65 ± 0.15	0.856 ± 0.012	1.42 ± 0.17	203.3 ± 7.28	90.54 ± 0.06
S2—medium	6.79 ± 0.24	0.825 ± 0.015	1.37 ± 0.20	190.6 ± 5.26	80.19 ± 0.01
S3—dark	7.92 ± 0.19	0.794 ± 0.016	1.44 ± 0.19	134.7 ± 4.09	71.24 ± 0.02
S4—pale	7.54 ± 0.27	0.810 ± 0.024	1.70 ± 0.13	181.5 ± 6.10	72.92 ± 0.07
S5—medium	7.81 ± 0.22	0.832 ± 0.008	1.39 ± 0.09	169.1 ± 8.39	69.23 ± 0.04

S1–S5—the aqueous extracts of the propolis samples; GAE—gallic acid equivalents; QE—quercetin equivalents.

**Table 2:** Diameters of the inhibition zones of the microbial strains for the aqueous extracts of propolis and ciprofloxacin.

	Sample No.	Diameters of Inhibition Zones (mm)						
Strain		S1 (0.1 g/mL)	S2 (0.1 g/mL)	S3 (0.1 g/mL)	S4 (0.1 g/mL)	S5 (0.1 g/mL)	Average x_j_	Ciprofloxacin (5 µg)
ATCC 49226		40	39	41	38	33	38.2	41
NG I		42	37	36	35	30	36	41
NG II		40	41	38	39	33	38.2	35
NG III		37	36	30	31	27	32.2	20
Average x_i_		39.75	38.25	36.25	35.75	30.75	∑x_ij_ = 723	-

S1–S5—the aqueous extracts of the propolis samples.

**Table 3:** The minimum inhibitory concentration (MIC) of various propolis types.

Sample No.	MIC (µg/mL)
S1	6.25
S2	6.25
S3	12.5
S4	6.25
S5	25.0

**Table 4:** Statistical bifactorial variance analysis for propolis samples.

Dispersion Sum of the Diameters of Inhibition Zones	Quadratic Sum	Degrees of Freedom ν	Variance	F_computed_	F_0.05_
Between the propolis types	S_2_–S_4_ = 186.80	m − 1 = 4	s_1_^2^ = 46.70	15.47	3.26
Between strains	S_3_–S_4_ = 120.15	n − 1 = 3	s_2_^2^ = 40.05	13.50	3.4 + 9
Residual, S_r_	S_r_ = 198	(m − 1)(n − 1) = 12	s_r_^2^ = 2.96	-	-

**Table 5:** The sequences of the *gyrA*, *parC*, and *dcmH* gene primers.

QRDR Primer	Sequence	Product Length	GC%
*gyrA*		391	
Forward	ATGTGAGATTTTCGCCATGCGG		62.14
Reverse	CAAATTCGCCCTCGAAACCCT		61.22
*parC*		329	
Forward	CAGCGGCGCATTTTGTTTG		59.51
Reverse	TCAAACGCGCCGTCGTAG		60.80
*dcmH*		80	
Forward	GGATACGACGTAACCTTGACTATGG		60.79
Reverse	CCGATGTAGAAGACCCTTTTGC		59.32

## Data Availability

Data are contained within the article.
